# Can Age-Friendly Planning Promote Equity in Community Health Across the Rural-Urban Divide in the US?

**DOI:** 10.3390/ijerph17041275

**Published:** 2020-02-17

**Authors:** Xue Zhang, Mildred E. Warner, Elaine Wethington

**Affiliations:** 1 Department of City and Regional Planning, Cornell University, Ithaca, NY 14853, USA; xz435@cornell.edu; 2 Department of Human Development, Roybal Center, Cornell University, Ithaca, NY 14853, USA; ew20@cornell.edu

**Keywords:** community health, rural–urban divide, livability, age-friendly planning, social and built environment

## Abstract

In the US, rural communities face challenges to meet the community health needs of older adults and children. Meanwhile, rural areas lag in age-friendly built environment and services. AARP, a US based organization promoting livability for all ages, has developed a Livability Index based on the World Health Organization’s (WHO) domains of age-friendly communities: health, housing, neighborhood, transportation, environment, engagement, and opportunity. This study links the 2018 AARP Livability Index categories with demographic structure and socio-economic factors from the American Community Survey at the county level in the US to examine if the physical, built and social environment differentiate communities with better community health across the rural–urban divide. Results show that the neighborhood built environment has the largest impact on community health for all county types. Although rural areas lag in community health, those which give more attention to engagement and opportunity rank higher. Rural communities with more African Americans, children, and poor Whites, rank lower on community health. While neighborhood characteristics have the strongest link to community health, a broader approach with attention to age, race, poverty and engagement and opportunity is needed for rural areas.

## 1. Introduction

Health disparities are not only related to race, gender, income, and education at the individual level, but also are a place-based issue related to geographic location, neighborhood, built environment, and community service delivery [1,2,3]. The World Health Organization (WHO) has articulated key domains for evaluating age-friendly communities across the world [4]. WHO’s age-friendly domains, based on extensive research, include the physical and built environment, social environment and services, and civic engagement [4]. WHO’s domains are consistent with the child-friendly recommendations promoted by the United Nations Children’s Fund (UNICEF) [5]. This overlap illustrates the potential for attention to age-friendly domains to increase community health for both older adults and children [6].

In the US, the AARP [7] and the American Planning Association [8] are leading efforts to promote child and age friendy communities, based on the dimensions set out by WHO [4] and UNICEF [5]. AARP leads the US in WHO’s Global Network of Age-Friendly Cities and Communities. In 2014, AARP developed a Livability Index based on WHO’s age-friendly domains and their own policy research on livable community principles (http://www.aarp.org/policybook) [4,7,9]. The seven domains included in the AARP livability categories are: health, housing, neighborhood, transportation, environment, engagement, and opportunity.

This paper examines community health disparities from an all-age lens and a rural–urban lens. In the US, rural communities face more challenges than urban and suburban areas in reaching health equity [3,10]. Rural areas are home to a disproportionate share of the elderly in the US [11] and around the world [12]. In the US, rural areas also attract poor and minority young families with children [13,14]. Ethnic and class differences between the younger and older populations contribute to the health disparities in US rural communities, as well as community health disparities between rural and urban areas [2,3]. In the US, rural African American children suffer the highest morbidity in obesity, compared to urban children [3,15]. However, US rural areas lag in health care infrastructure and services [3,16,17]. Lack of access to health care in US rural communities is related to poor health outcomes and high cost [18].

Age-friendly approaches to community planning in the US are being used to promote community health [8]. An age-friendly community aims to increase functional independence for individuals across the life course by linking individual physical capacity, the built environment (determined by planning and zoning), and service delivery in the community [19,20]. However, design guidelines adhering to new urbanist principles of density, walkability, and mixed-use [21,22] are difficult for rural communities to implement due to lower density and greater dependence on automobile travel [19,20]. Rural communities face challenges with the built environment and the services needed to meet the health needs of older adults and children.

In this study, we are interested in the factors differentiating health outcomes, and the relation between community health, the physical, built and social environment, and socio-economic conditions across the rural–urban divide. This paper uses the AARP Livability Index at the US county level to explore rural–urban differences in health, physical and built environment (housing, neighborhood, transportation), and social environment (engagement, and opportunity). This paper is the first to link the AARP livability categories with US Census data to examine factors differentiating community health in rural and urban settings.

## 2. Literature Review

Health disparities are partly a place-based issue embedded in the rural–urban divide [1,2]. This is especially the case for countries with sprawling and remote rural areas, such as Australia [18,23], Canada [12], and the US [2,19,24]. In the US, health disparities in rural communities are related to racial inequity, residential segregation and poverty [14,18], and income inequality [25,26]. For example, Taylor [3] explored the relation between rural health and economic indicators at the county level in the US, including education, employment, poverty and income inequity, and found that rural health disparities in heart disease, disability, and mortality rate (especially for children under age five) are related to geographic resource limitations such as lack of education and job opportunity, and limited access to healthcare services. Sameem and Sylwester [27] examined mortality rates between US urban and rural counties from 1990 to 2009, and found that rural counties have higher mortality rates after controlling for age, race, and gender. Lutfiyya et al. [28] analyzed the prevalence of obese children between rural and urban areas from 2003 to 2004, and found that rural residency is an independent risk factor for childhood obesity in the US.

The built environment (e.g., neighborhood, housing, transportation) impacts health disparities [29,30]. In the US, rising rates of obesity among children [28,31] and older adults [32] have led to calls for increased attention to opportunities for physical activity in streetscape design and park development [33,34,35]. For example, a meta-analysis [36] reported an association between planning, transportation, and physical environment (such as parks and trails), on adults’ and children’s physical activity. In US nonmetropolitan communities, more outdoor recreation facilities are related to a lower prevalence of obesity among both children [37], and adults [38]. Even a state level variable, such as suburban sprawl, has been associated with the rise in obesity in the US since the 1990s [39]. Satariano et al. [40] reviewed studies on mobility and aging, and emphasized the importance of mixed-used neighborhoods and complete streets in encouraging walking and safer driving of older adults. Giles-Corti et al. [41] found that street safety and the proximity to local destinations are determinants of children’s independent mobility.

However, it is difficult to build a better physical built environment to promote community health for older adults and children in rural communities. For example, projects on urban green space [42], and compact cities [43] are urban-focused. In the US, rural areas face greater challenges to building walkable, mixed-use environments, due to less dense development patterns. Rural communities lag in child- and age-friendly built environment and services [17,44], despite the fact that rural and suburban communities have a higher percentage of older adults [17] and young minority families with children [13,14]. Urban studies focus on the inadequacy of park space in low income and minority neighborhoods in the US [45,46]. Rural communities also face problems with the accessibility and safety of public parks [37]. For rural communities, transportation is often highlighted as the biggest challenge [47].

The social environment also matters for rural community health. A review of the global research finds that community participation helps rural communities deal with health issues such as accessibility and equity [48]. Ochoa and Nash [49] reviewed community-based projects addressing health disparities in the US, and concluded that community engagement affects childhood disparities in diabetes and other risk factors. Studies of US communities found those which engaged older adults in the planning process were more likely to provide health related services [17], and those which engage elders and families with children were more likely to have age friendly planning and design [19,20,44].

## 3. Method

### 3.1. Research Questions

This study uses the US-based AARP Livability Index to assess the relationship between the physical, built and social environment, socio-economic conditions and community health across 3139 US counties. Three research questions are addressed: (1) How do community health, physical and built environment, and social environment differ across the rural–urban divide? (2) Is the relationship between community health and social and built environment differentiated by metro status? (3) Do differences in community age and race composition differentiate community health across urban and rural areas?

### 3.2. Data

This study uses AARP’s 2018 Livability Index community health category as the outcome variable. Independent variables include AARP’s 2018 categories for the physical, built and social environment. Variables regarding demographic structure and socio-economic data are from the American Community Survey most recent estimates (2013–2017). All variables are at the county level.

The AARP Livability Index measures WHO’s age-friendly domains including seven categories: health, physical environment, neighborhood, housing, transportation, engagement, and opportunity. The AARP livability categories were developed by the AARP Public Policy Institute (PPI). The AARP PPI team used a mixed-methods exploratory sequential design to develop a set of principles about the factors that older adults think about when assessing the livability of a community [50]. AARP conducted focus groups, interviews with key informants and a national livability survey of about 5000 individuals age 50 and over in 2012 to understand preferences regarding livability. PPI integrated and interpreted the data to develop objective indicators of community factors associated with livability. After identifying the seven categories, the AARP PPI identified nationally public and private available sources of data that could provide metrics to measure these categories. In all, 40 metrics were chosen; most measured at the neighborhood level. The AARP PPI aggregated the metrics to the county level and averaged them to form the seven categories. Metrics within each category are weighted equally. Each category is standardized on a scale from 0 to 100, and the average community has an average total score of 50 [7]. For more detail on the construction of the seven categories, see the Appendix A. There is no publicly available data set of the AARP sub-metrics. AARP only made available the seven overall category values at the county level to the authors for this study.

### 3.3. Measures

#### 3.3.1. Outcome Variable: Community Health

Community health is measured by the health category in the AARP Livability Index and captures prevention, access and quality. It aggregates metrics from three subcategories: healthy behaviors (smoking prevalence, obesity prevalence, access to exercise opportunities), access to health care (health care professional shortage areas), and quality of health care (preventable hospitalization rate, patient satisfaction). Healthy behaviors are measured at the county level, but access to health care and quality of health care are measured at the regional level (the health professional shortage area and hospital service area, respectively). The US national average score of health is 40, and the standard deviation is 13.61.

#### 3.3.2. Independent Variables

##### Physical, Built and Social Environment

The physical and built environment categories address pollution, neighborhood, housing and transportation. *Environment* measures clean air and water, including drinking water quality, regional air quality, near-roadway pollution, and local industrial pollution, all aggregated to the county level. *Neighborhood* includes proximity to destinations (access to grocery stores and farmers’ markets, access to parks, access to libraries, access to jobs by transit, access to jobs by automobile), mixed-use neighborhoods (diversity of destinations), compact neighborhoods (activity density), personal safety (crime rate), and neighborhood quality (vacancy rate) all aggregated to the county level. *Housing* includes three subcategories: housing accessibility (zero-step entrances, calculated at the metro level), housing options (availability of multi-family housing), and housing affordability (housing costs, housing cost burden, availability of subsidized housing). All sub-metrics are aggregated to the county level for a single overall housing score. *Transportation* includes accessible system design (American with Disabilities Act (ADA)-accessible stations and vehicles, calculated at the metro level), convenient transportation options (walk trips, congestion, frequency of local transit service), transportation costs (household transportation costs, calculated at the metro level), and safe streets (speed limits, crash rate). All metrics are aggregated to the county level for a single overall transportation score.

The social environment includes engagement and opportunity. *Engagement* includes internet access (broadband cost and speed), civic engagement (voting rate), and social engagement (cultural, arts, and entertainment institutions calculated at the metro level). All measures are aggregated to the county level and averaged to create a single category value for each county. *Opportunity* measures equal opportunity (income inequality), economic opportunity (jobs per worker, calculated at the metro level), educational opportunity (high school graduation rate), and multi-generational communities (age diversity). All metrics are aggregated to the county level for a single overall opportunity score.

These six county level categories explore which factors in the physical, built and social environment differentiate community health in metro core, suburb, micro core and remote rural counties.

##### Socio-Economic Conditions

Demographic structure and economic variables are drawn from American Community Survey (ACS 2013–2017) data on age, race, income and inequality at the county level. Race/ethnicity is differentiated by percent non-Hispanic Black, percent Hispanic, and percent non-Hispanic Other (American Indian, Asian, Native Hawaiian, two or more other races). Non-Hispanic White is set as the reference group. To capture the proportion of children and older adults in each county, the percent of population under 18 and percent of population over 65 are used. Education is percent of population over age 25 with a bachelor’s degree or higher, and economic variables include percent of households with retirement income, per capita income, percent of poverty rate in the non-Hispanic White population, and the Gini coefficient of income inequality. The models also control for median age of housing, population, population density, and population growth.

##### Metro Status

Counties are classified into four groups by metro status. Classification is based on core-based statistical area and principal city defined by the Office of Management and Budget in 2018 (https://www.census.gov/geographies/reference-files/time-series/demo/metro-micro/delineation-files.html). Metro core are counties with at least one principal city inside a metropolitan area. Suburbs are other counties inside metropolitan areas. Micro core are counties in non-metropolitan areas which have at least one principal city. Remote rural are all other nonmetropolitan counties.

### 3.4. Analysis

This study uses the most recent 2018 AARP Livability Index and Census data to assess the relationship between physical, built and social environment, socio-economic conditions and health across US counties. To address Research Question 1, the Scheffé test for differences in subgroup means is used to examine rural–urban differences in the AARP livability categories. To address Research Questions 2 and 3, ordinary least squares (OLS) regression is used to differentiate community health by the AARP livability categories and metro status (metro core, suburb, micro core and remote rural) (Research Question 2), and by age and ethnicity (Research Question 3). A positive relation between community health and the other AARP livability categories is expected, but the impact of each livability category on community health is expected to vary by metro status. Because health disparity is a place-based issue, differences related to demographic structure and socioeconomic conditions are also expected.

## 4. Results

Table 1 shows the descriptive statistics and Scheffé test results for all model variables to examine rural–urban differences (Research Question 1). Results show statistically significant differences in community health among metro core, suburb, micro core, and remote rural counties. The outcome variable. Health shows a clear gradient with the highest value in metro (${\bar{x}}_{metro}:$ 50), lower in suburbs (${\bar{x}}_{suburb}:$ 41), lower in micro core (${\bar{x}}_{micro}:$ 40) and lowest in remote rural (${\bar{x}}_{remote\;rural}:$ 35). This confirms the expectation that rural areas lag on community health. Remote rural counties also significantly lag on neighborhood (${\bar{x}}_{metro}:$ 46, ${\bar{x}}_{suburb}:$ 39, ${\bar{x}}_{micro}:$ 38, ${\bar{x}}_{remote\;rural}:$ 34), and opportunity (${\bar{x}}_{suburb}:$ 60, ${\bar{x}}_{metro}:$ 53, ${\bar{x}}_{micro}:$ 51, ${\bar{x}}_{remote\;rural}:$ 42) compared to all other county types. Environment shows no difference across groups. Remote rural counties have the highest score on housing because they have more affordability. Interestingly, remote rural counties rank highest on engagement compared to all other county types (${\bar{x}}_{remote\;rural}:$ 57, ${\bar{x}}_{suburb}:\;$55, ${\bar{x}}_{metro}:\;$53, ${\bar{x}}_{micro}:$ 52). Metro core counties rank highest on neighborhood (${\bar{x}}_{metro}:$ 46, ${\bar{x}}_{suburb}:\;39,\;{\bar{\;x}}_{micro}:$ 38, ${\bar{x}}_{remote\;rural}:$ 34), and transportation (${\bar{x}}_{metro}:$ 50, ${\bar{x}}_{micro}:$ 47, ${\bar{x}}_{remote\;rural}:$ 45, ${\bar{x}}_{suburb}:$ 43) as expected. Suburban counties have the highest opportunity score (${\bar{x}}_{suburb}:$ 60), and the lowest transportation score (${\bar{x}}_{suburb}:\;$43). Micro core counties rank the second highest on housing (${\bar{x}}_{micro}:\;$56) and transportation (${\bar{x}}_{micro}:\;$47). Demographic structure shows that minority groups are concentrated in the metro core. Remote rural areas have a lower percentage of children (22%), a higher proportion of older adults (20%), and a lower proportion of adults with a higher degree (18%). Regarding socio-economic conditions, micro core and remote rural areas have lower income, higher poverty and more older housing stock.

Ordinary Least Squares (OLS) regressions are used to address Research Questions 2 and 3. Both P-P plot and multicollinearity tests verify the appropriateness of using of OLS: residuals are normally distributed and Variance Inflation Factor (VIF) is between 2.16 and 4.03. Model results are shown in Table 2.

Coefficients associated with the AARP livability categories address Research Question 2: the built and social environment categories have different effects on community health, and these effects vary across the rural–urban divide. The overall model results show that both suburbs and remote rural areas lag in community health (Table 2, Column 1). Neighborhood has the highest impact on community health of all the livability categories for all county types. The sub-models by metro status show that neighborhood is the only category that has a significant impact in metro core counties (β = 0.42). For suburbs, neighborhood, transportation and engagement are significant, but neighborhood (β = 0.29) has twice the effect of transportation (β = 0.15), and three times the effect of engagement (β = 0.10).

For rural counties (micro core and remote rural), a broader set of livability categories have a statistically significant impact on community health. While neighborhood has the largest effect, transportation, engagement and opportunity also have positive impacts. In micro core counties, community health is positively related to neighborhood (β = 0.27), engagement (β = 0.10) and opportunity (β = 0.08). Better community health in remote rural counties is also related to better neighborhood (β = 0.20), transportation (β = 0.07), engagement (β = 0.04), and opportunity (β = 0.09). Housing is negatively associated with health for micro core (β = −0.50) and remote rural counties (β = −0.51). The housing variable is heavily weighted toward affordability and thus housing is positively correlated with poverty (r = 0.36). Poverty is negatively correlated with community health (r = −0.53), which may explain the housing result.

Coefficients associated with demographic structure and socio-economic conditions address Research Question 3. Race, age group and population density differentiate community health across metro status.

Race matters: Counties with more non-Hispanic Blacks rank lower on community health, for all county types, and counties with more population of other races also rank lower on health in the overall model and in the remote rural model. By contrast, counties with more Hispanics rank higher on health, across all county types. This “Hispanic paradox” will be discussed in more detail in the discussion.

Income matters: Although health is better in higher income counties, income does not differentiate community health by metro status. The differentiation comes in the composition of income and population. Counties with more households with retirement income (β = −0.11) and more poverty (β = −0.11) have lower health in the overall model. But the sub-models show these results are primarily related to rurality. Health is lower in micro core counties with more retirement income (β = −0.24) and in remote rural counties with more non-Hispanic White poverty (β = −0.14). Counties with greater inequality (Gini) have lower health in the overall model (β = −26) and in the suburban model (β = −43).

Age matters, with lower health in counties with more children and higher health in counties with more older adults in the Overall model (Column 1). This effect is primarily due to the remote rural counties, where those with more children have lower health outcomes (β = −0.25), and those with more older adults have higher health outcomes (β = 0.44).

Education is positively associated with health in all county types, as expected. Age of housing is negatively associated with health in the overall model (β = −0.06) and in the micro core (β = −0.10).

Another factor linking livability and health is population density. While overall model results show counties with larger population have higher community health (β = 0.53), this is only true for metro core counties (β = 0.92). Population density is negative overall (β = −0.71), and in the metro core (β = −1.58) and micro core (β = −1.02) models, which raises questions about the role of density alone on community health. Population growth does not differentiate county health.

## 5. Discussion

This study gives fresh insights into how to understand livability across metro status. The first research question examines rural–urban differences in the AARP livability categories and finds rural counties (both micro core and remote rural) lag in health and in the built environment. Neighborhood characteristics matter for health, but these built environment features (walkability, mixed use, proximity) are most likely to be found in the metro core.

Regression model results show the different impact of built and social environment on community health (Research Question 2). For metro core counties, only neighborhood is related to community health. For suburbs and remote rural areas attention must be given to a broader set of factors. Model results suggest work on the neighborhood environment will have more impact on suburban community health than transportation, however, transportation is linked to land use, as suburbs are characterized by sprawl. Engagement also has a positive effect.

In rural areas (both micro core and remote rural), opportunity and engagement also differentiate counties with higher health outcomes. These results show the importance of a broader approach to livability for rural counties. Built environment matters for health, but neighborhood measures are urban biased, and these built environment features are unrealistic for most rural areas. The fact that engagement, opportunity and transportation all have positive impacts on health shows the possibility for rural-focused strategies more specifically targeted to the unique environments of rural communities.

Remote rural counties rank the highest in engagement (Table 1). Prior research has confirmed the critical importance of community engagement in building more child and age friendly communities [17,19,20,44,51,52]. Both WHO [4,9], UNICEF [5] and the American Planning Association (APA) [8,53] point to the need to give attention to the social environment—economic opportunity and engagement—beyond the built environment features of housing, neighborhood and transportation.

Research shows engagement and opportunity play an important role in community health [48,49], and this study finds this is especially true for rural communities. Civic engagement helps rural communities enhance access to health services [17,48]. Public health promotion interventions can focus on community services that enhance or extend access—such as paratransit or informal services. While emphasis on neighborhood and transportation characterizes most age friendly research, this study suggests that more research is needed on the effect of opportunity and engagement on community health, especially for rural communities.

This study shows race and income composition differentiate community health across the rural–urban divide (Research Question 3). Counties with more African American population rank lower on health across all county types. This was expected, as communities with more African American population have lower health outcomes [37] and lower life expectancy [54]. However, model results show two surprises: counties with more Hispanics have higher community health across all county types, and counties with more White poverty have lower health. The positive effect of Hispanics is referred to as the “Hispanic paradox” in health, which is as yet not fully explained [55]. Health benefits start young, and it could be related to more access to fresh fruit and vegetables [24], and lower tobacco use [56] in Hispanic communities. The negative impact of non-Hispanic White poverty on health shows that the negative impact of poverty on health is more severe with the increase of rurality. Income and population composition differentiate community health, and we find lower health in suburban communities with more inequality. As suburban counties have become more diverse, by race and income, more attention is being given to the planning and health challenges of suburbs [14,57]. Community health is lower in micro core counties with more households with retirement income, and in remote rural counties with more non-Hispanic White poverty. Greater attention needs to be given to the composition of rural retirees [11], and the challenges of poverty in remote rural counties [58].

Age composition also matters. Counties with more children have lower health overall, but this factor primarily differentiates remote rural counties. Other research has found rates of child obesity are higher in rural counties [28,31]. While rural community health is negatively related to the percentage of children, the positive link between community health and the percentage of older adults may be partially explained by in-migration of healthy retirees, especially in retirement destination rural counties [11]. The model results paint a disturbing picture of remote rural counties with lower health status where African Americans, poor Whites and young children constitute a larger share of the population. Pruitt [59] argues that community wellbeing needs a racial and class lens, because negative stereotypes lead to reduced social investment in both communities with poor African Americans and poor Whites. Remote rural counties with more children have lower community health, but those with a higher proportion of older adults have higher health. This raises the importance of age-friendly planning to meet the needs of both older adults and children across the life course.

This research points to the critical role of community planning to build environments more conducive to health. Population density alone does not make the difference, as population density is associated with lower health in more dense counties, primarily in the metro core and micro core counties. Density must be accompanied by neighborhood characteristics (mixed use, walkability) to make it positive. Zoning codes can create the built environments that best meet the needs of children [44] and older adults [20], if special attention is given to participation of those most affected—children, the poor and older adults [19,20,44]. In rural areas, where the built environment is least amenable to change, services must make up the difference, but prior research has shown health related services also lag in rural areas [17,60]. Rural local governments face the challenges of urban-biased design guidelines and the lack of infrastructure and services [19]. Health is a place-based issue, and collaboration between residents, business and community institutions is essential to improve public health [1], especially in rural communities [20].

This study has several limitations. First, although the AARP livability categories cover all counties in the US, 5 out of the 40 sub-metrics are only available at the metro area level. AARP recognizes the data limitations [7], and imputation information is provided in the Appendix A to this paper. The results on engagement need to be treated with caution, because one of the five component metrics, the social involvement index, is measured at the metro area, and the missing values are imputed from the state average. All remote rural counties in the same state could get the same number. However, this sub-metric only contributes 20% of the engagement score. The engagement category is normally distributed for each metro status, and still captures the rural–urban divide. Second, this study builds the connection between health, built environment, demographic structure and socio-economic conditions, but the role of government policy is not measured. Previous research shows that communities with comprehensive plans addressing the needs of older adults and children have more age-friendly zoning codes and built environments [20,44]. Future research could explore the impact of community level planning on community health. Third, the AARP health category includes healthy behavior, access to health care, and quality of health care at the county level. Because this study focuses on county level indicators, the impact of social and physical environment on individual health outcomes is not measured. Future study could use other health datasets to get individual level data, such as datasets from the National Center for Health Statistics, and Agency for Healthcare Research and Quality.

## 6. Conclusions

This is the first study to link the unique US-based AARP Livability Index and US census data to explore the relation between community health and the physical, built and social environment, demographic structure and social-economic conditions across the rural–urban divide. Community health studies need to broaden their framework to include attention to both the built and social environment.

The impact of the built and social environment on health varies across metro status. Neighborhood is the only category significantly related to community health in metro core counties. For the more rural counties, more livability categories are related to community health. Health is a place-based issue and more attention needs to be paid to age-friendly design guidelines, planning, local government policy, and service delivery, especially in rural counties.

Age, race and income show different effects on health across the rural–urban divide. It is important to look at the composition of income and population to understand differences in community health across metro status. For example, suburbs with greater income inequity have lower community health, and remote rural counties with more African Americans, more children and more White poverty lag in community health, compared to other counties. It is important to look across age, race and rurality to develop strategies to help communities promote community health.

A key finding of this research is that rural counties, which give more attention to creating an inclusive environment, rank higher on health. Local government actions to build age-friendly communities are important. Planning, which can increase civic engagement and opportunities, has a key role to play in helping move rural communities forward in addressing public health challenges and creating livable communities for all ages [20,44]. A comprehensive approach must link the built environment, with community health and recognize the critical importance of social environment (public engagement and opportunity). It is the balance between built environment, services and community engagement and opportunity that makes the difference for rural community health. Engagement and opportunity represent the social layer that needs more attention, especially for rural communities.

## Figures and Tables

**Table 1:** Descriptive statistics, US counties (mean).

	All	Metro Core	Suburb	Micro Core	Remote Rural
**AARP Livability Category**					
Health (0–100) ^a^	39.86	50.17 ^1^	41.18 ^2^	39.64 ^3^	35.27 ^4^
Environment (0–100) ^a^	52.47	53.30 ^1^	52.46 ^1^	51.96 ^1^	52.34 ^1^
Neighborhood (0–100) ^a^	37.81	46.45 ^1^	39.12 ^2^	37.54 ^3^	33.90 ^4^
Housing (0–100) ^a^	55.29	51.46 ^3^	51.59 ^3^	55.86 ^2^	58.22 ^1^
Transportation (0–100) ^a^	45.81	50.29 ^1^	42.65 ^4^	47.29 ^2^	44.83 ^3^
Engagement (0–100) ^a^	54.99	52.88 ^3^	55.00 ^2^	51.72 ^3^	57.10 ^1^
Opportunity (0–100) ^a^	49.07	53.22 ^2^	60.24 ^1^	51.00 ^2^	41.78 ^3^
**Demographic Structure**					
Percent of non-Hispanic Black (%) ^b^	8.90	11.59 ^1^	10.75 ^1^	8.22 ^2^	7.29 ^2^
Percent of non-Hispanic Other (%) ^b^	5.15	6.88 ^1^	3.83 ^3^	4.73 ^2,3^	5.19 ^2^
Percent of Hispanic (%) ^b^	9.13	13.49 ^1^	6.33 ^2^	11.22 ^1^	7.80 ^2^
Percent of population under 18 (%) ^b^	22.46	22.82 ^1^	22.80 ^1^	22.73 ^1^	22.07 ^2^
Percent of population over 65 (%) ^b^	17.95	15.11 ^3^	16.65 ^2^	16.99 ^2^	20.02 ^1^
Percent of population over age 25 with at least a bachelor degree (%) ^b^	21.22	29.62 ^1^	22.63 ^2^	20.44 ^3^	17.55 ^4^
**Socio-Economic Condition**					
Percent of households with retirement income (%) ^b^	20.05	19.46 ^2^	21.51 ^1^	19.92 ^2^	19.71 ^2^
Per capita income (ln) ^b^	10.14	10.27 ^1^	10.21 ^2^	10.09 ^3^	10.08 ^3^
Poverty, non-Hispanic White pop (%) ^b^	12.73	10.86 ^2^	11.09 ^2^	13.88 ^1^	13.75 ^1^
Gini Coefficient of Income Inequality (0–1) ^b^	0.44	0.46 ^1^	0.43 ^3^	0.45 ^2^	0.44 ^2^
Median age of housing (yrs) ^b^	41.84	39.49 ^3^	36.90 ^4^	42.17 ^2^	44.80 ^1^
Population (ln) ^b^	10.27	12.28 ^1^	10.60 ^2^	10.64 ^2^	9.18 ^3^
Population density (ln) ^b^	3.78	5.81 ^1^	4.51 ^2^	3.98 ^3^	2.58 ^4^
Population growth (%) ^b,c^	0.56	4.06 ^1^	1.60 ^2^	0.32 ^3^	−1.20 ^4^
N	3139	563	617	548	1411

Superscript numbers, represent Scheffé post hoc rankings (^1^ = highest ranking, ^2^ = medium high ranking, ^3^ = lower ranking, ^4^ = lowest ranking, based on alpha = 0.05). Data sources: ^a^ AARP Livability Index 2018, ^b^ American Community Survey 2013–2017, ^c^ American Community Survey 2008–2012.

**Table 2:** Differentiating Community Health by Metro Status and Livability Categories: OLS model results (US counties).

	Overall		Metro Core		Suburb		Micro Core		Remote Rural	
	Coef.	S.E.	Coef.	S.E.	Coef.	S.E.	Coef.	S.E.	Coef.	S.E.
**AARP Livability Category**										
Environment (0–100) ^a^	−0.01	(−0.80)	−0.00	(−0.10)	−0.02	(−0.80)	0.02	(0.65)	−0.00	(−0.18)
Neighborhood (0–100) ^a^	0.24 **	(7.03)	0.42 **	(5.28)	0.29 **	(3.92)	0.27 **	(2.68)	0.20 **	(3.96)
Housing (0–100) ^a^	−0.22 **	(−6.28)	−0.08	(−1.12)	−0.05	(−0.84)	−0.50 **	(−4.29)	−0.51 **	(−7.76)
Transportation (0–100) ^a^	0.09 **	(4.17)	−0.00	(−0.09)	0.15 **	(3.53)	0.14	(1.81)	0.07 *	(2.30)
Engagement (0–100) ^a^	0.06 **	(4.59)	0.03	(0.94)	0.10 **	(3.70)	0.10 **	(2.95)	0.04 *	(1.98)
Opportunity (0–100) ^a^	0.06 **	(4.44)	−0.03	(−0.79)	0.01	(0.46)	0.08 *	(2.46)	0.09 **	(4.47)
**Demographic Structure**										
Percent of non-Hispanic Black (%) ^b^	−0.12 **	(−7.82)	−0.14 **	(−3.50)	−0.08 **	(−2.88)	−0.16 **	(−4.03)	−0.13 **	(−5.54)
Percent of non-Hispanic Other (%) ^b^	−0.07 **	(−3.65)	0.15 **	(2.78)	−0.15	(−1.56)	−0.01	(−0.16)	−0.07 *	(−2.53)
Percent of Hispanic (%) ^b^	0.15 **	(11.07)	0.08 *	(2.22)	0.19 **	(5.20)	0.09 **	(3.00)	0.15 **	(7.06)
Percent of population under 18 (%) ^b^	−0.22 **	(−3.68)	0.07	(0.42)	−0.00	(−0.03)	−0.20	(−1.24)	−0.25 **	(−2.84)
Percent of population over 65 (%) ^b^	0.28 **	(5.00)	0.09	(0.64)	0.15	(1.09)	0.26	(1.74)	0.44 **	(5.14)
Percent of population over age 25 with at least a bachelor degree (%) ^b^	0.74 **	(25.13)	0.69 **	(10.13)	0.71 **	(10.75)	0.71 **	(9.59)	0.74 **	(14.31)
**Socio-Economic Condition**										
Percent of households with retirement income (%) ^b^	−0.11 **	(−2.78)	−0.18	(−1.52)	−0.15	(−1.82)	−0.24 *	(−2.29)	−0.06	(−1.06)
Per capita income (ln) ^b^	2.90 *	(1.97)	0.65	(0.15)	3.08	(0.87)	4.68	(1.10)	2.21	(1.04)
Poverty, non-Hispanic White pop. (%) ^b^	−0.11 *	(−2.19)	−0.06	(−0.34)	0.00	(0.01)	−0.27	(−1.87)	−0.14 *	(−2.18)
Gini Coefficient of Income Inequality (0–1) ^b^	−25.94 **	(−4.43)	−36.48	(−1.94)	−43.15 **	(−3.49)	3.16	(0.19)	−7.59	(−0.89)
Median age of housing (yrs) ^b^	−0.06 **	(−3.04)	−0.05	(−0.98)	−0.08	(−1.96)	−0.10 *	(−2.08)	0.02	(0.72)
Population (ln) ^b^	0.53 *	(2.25)	0.92 *	(2.08)	0.10	(0.27)	−0.18	(−0.22)	0.16	(0.39)
Population density (ln) ^b^	−0.71 **	(−3.74)	−1.58 **	(−3.79)	−0.42	(−1.09)	−1.02 *	(−2.08)	−0.41	(−1.28)
Population growth (%) ^b,c^	0.01	(0.16)	0.03	(0.29)	0.03	(0.28)	−0.18	(−1.55)	0.01	(0.26)
**Metro Status (Reference = Metro Core)**										
Suburb	−2.04 **	(−3.62)								
Micro core	−0.55	(−1.00)								
Remote rural	−1.74 **	(−2.76)								
Constants	4.36	(0.28)	24.60	(0.52)	−3.93	(−0.11)	−3.02	(−0.07)	15.77	(0.71)
N	3139		563		617		548		1411	
Adjusted R square	0.67		0.72		0.74		0.67		0.54	

Author Analysis of US Counties, Data sources: ^a^ AARP Livability Index 2018, ^b^ American Community Survey 2013–2017, ^c^ American Community Survey 2008–2012. Note: ** *p* < 0.01, * *p* < 0.05, unstandardized coefficients.

## Appendix A. Construction of the AARP Livability Categories

The seven AARP livability categories were developed by AARP Public Policy Institute (PPI) and are built up from 40 individual metrics. The data source for each metric can be viewed at https://livabilityindex.aarp.org/livability-sources. Most metrics are measured at the neighborhood level, which are then aggregated to the county level and then averaged to form the seven categories (Table A1). Metrics at the metro area level used the following imputation rules: (1) the missing values of “zero-step entrances (housing)” and “jobs per worker (opportunity)” are replaced by the US mean. (2) The missing values of “ADA-accessible stations and vehicles (transportation)” for rural areas are replaced by the rural average calculated from the data submitted by rural transit agencies. (3) The missing values of “congestion (transportation)" are substituted by zero. (4) The missing values of “social involvement index (engagement)” are substituted by the state average.

**Table A1:** Component Metrics of AARP Livability Index Categories.

Category	Component Metric	Measurement	Units	Scale
Health prevention, access and quality	Healthy behaviors	Smoking prevalence	Pct. of people who smoke regularly	County
	Healthy behaviors	Obesity prevalence	Pct. of people who are obese	County
	Healthy behaviors	Access to exercise opportunities	Pct. of people with access (within 0.5 mile of parks, 3 miles for rural areas)	County
	Access to health care	Health care professional shortage	Index from 0 to 25	Health professional shortage area
	Quality of health care	Preventable hospitalization rate	Preventable hospitalizations per 1000 patients	Hospital service area
	Quality of health care	Patient satisfaction	Pct. of patients giving hospital highest satisfaction rate	Hospital service area
Environment: clean air and water	Water quality	Drinking water quality	Pct. of people exposed to public water systems with health violations	County
	Air quality	Regional air quality	Unhealthy air quality days per year	County
	Air quality	Near-roadway pollution	Pct. of people living close to high traffic roads	Neighborhood
	Air quality	Local industrial pollution	Toxicity of airborne chemicals, index from 0 to 311,000	Neighborhood
Neighborhood access to life, work, and play	Proximity to destinations	Access to grocery stores and farmers’ markets	No. stores and markets within a half mile	Neighborhood
	Proximity to destinations	Access to parks	No. parks within a half mile	Neighborhood
	Proximity to destinations	Access to libraries	No. libraries within a half mile	Neighborhood
	Proximity to destinations	Access to jobs by transit	No. jobs within 45 minute transit commute	Neighborhood
	Proximity to destinations	Access to jobs by auto	No. jobs with 45 minutes automobile commute	Neighborhood
	Mixed-use neighborhoods	Diversity of destinations	Mix of jobs within a mile. Index from 0 to 1	Neighborhood
	Compact neighborhoods	Activity density	Jobs and people per sq. mile	Neighborhood
	Personal safety	Crime rate	Crimes per 10,000 people	County
	Neighborhood quality	Vacancy rate	Pct. of vacant units	Neighborhood
Housing affordability and access	Housing accessibility	Zero-step entrances	Pct. of units	Metro area
	Housing options	Availability of multi-family housing	Pct. of housing that is multi-family units	Neighborhood
	Housing affordability	Housing costs	Avr. monthy housing cost	Neighborhood
	Housing affordability	Housing cost burden	Pct. of income spent on housing	Neighborhood
	Housing affordability	Availability of subsidized housing	No. subsidized units per 10,000 people	Neighborhood
Transportation: safe and convenient options	Convenient transportation options	Frequency of local transit service	No. buses and trains per hour	Neighborhood
	Accessible system design	ADA-accessible stations and vehicles	Pct. of accessible stations and vehicles	Metro area
	Convenient transportation options	Walk trips	No. trips per household per day	Neighborhood
	Convenient transportation options	Congestion	No. hours spent commuting per person per year	Metro area
	Transportation costs	Household transportation costs	Total dollars spent per household per year	Neighborhood
	Safe streets	Speed limits	Avr. speed limit	Neighborhood
	Safe streets	Crash rate	Fatal crashes per 100,000 people per year	Neighborhood
Engagement: civic and social involvement	Internet access	Broadband cost and speed	Pct. of residents with access to high-speed, low-cost service	Neighborhood
	Civic engagement	Opportunity for civic involvement	No. civic, social, political, religious and business organizations per 10,000 people	County
	Civic engagement	Voting rate	Pct. of people over 18 years of age who voted	County
	Social engagement	Social involvement index	Index from 0 to 2 of family and neighborhood engagement	Metro area
	Social engagement	Cultural, arts, and entertainment institutions	No. cultural and sports institutions per 10,000 people	Neighborhood
Opportunity: inclusion and possibilities	Equal opportunity	Income inequality	Gini index, from 0 to 1	County
	Economic opportunity	Jobs per worker	No. jobs per worker	Metro area
	Educational opportunity	High school graduation rate	Pct. of students who graduate high school	School district
	Multi-generational communities	Age diversity	Index from 0 to 1	Neighborhood

Data Source: AARP Livability Index 2018, https://livabilityindex.aarp.org/ Compiled by author.
